# A TMT-Based Quantitative Proteome Analysis to Elucidate the TSWV Induced Signaling Cascade in Susceptible and Resistant Cultivars of *Solanum lycopersicum*

**DOI:** 10.3390/plants9030290

**Published:** 2020-02-26

**Authors:** Ravi Gupta, Cheol Woo Min, So Wun Kim, Ju Soon Yoo, Ah-Ram Moon, Ah-Young Shin, Suk-Yoon Kwon, Sun Tae Kim

**Affiliations:** 1 Department of Botany, School of Chemical and Life Sciences, Jamia Hamdard, Hamdard Nagar, New Delhi 110062, India; dr.ravigupta@jamiahamdard.ac.in; 2 Department of Plant Bioscience, Pusan National University, Miryang 627-707, Koreakimsso12@nate.com (S.W.K.); jsyoo71@hanmail.net (J.S.Y.); 3 Plant Systems Engineering Research Centre, Korea Research Institute of Bioscience and Biotechnology, Daejeon 34141, Korea; qaz_3627@naver.com (A.-R.M.); shinay@kribb.re.kr (A.-Y.S.); 4 Biosystems and Bioengineering Program, University of Science and Technology, Daejeon 34141, Korea

**Keywords:** TSWV, proteomics, tomato, plant–pathogen interaction, MAP kinase, signaling, tandem mass tags (TMT)

## Abstract

Tomato spotted wilt virus (TSWV), transmitted by small insects known as thrips, is one of the major threats to tomato productivity across the globe. In addition to tomato, this virus infects more than 1000 other plants belonging to 85 families and is a cause of serious concern. Very little, however, is known about the molecular mechanism of TSWV induced signaling in plants. Here, we used a tandem mass tags (TMT)-based quantitative proteome approach to investigate the protein profiles of tomato leaves of two cultivars (cv 2621 and 2689; susceptible and resistant to TSWV infection, respectively) following TSWV inoculation. This approach resulted in the identification of 5112 proteins of which 1022 showed significant changes in response to TSWV. While the proteome of resistant cultivar majorly remains unaltered, the proteome of susceptible cultivar showed distinct differences following TSWV inoculation. TSWV modulated proteins in tomato included those with functions previously implicated in plant defense including secondary metabolism, reactive oxygen species (ROS) detoxification, mitogen-activated protein (MAP) kinase signaling, calcium signaling and jasmonate biosynthesis, among others. Taken together, results reported here provide new insights into the TSWV induced signaling in tomato leaves and may be useful in the future to manage this deadly disease of plants.

## 1. Introduction

Tomato spotted wilt virus (TSWV), a member of the *Tospovirus* genus, is one of the major threats to tomato productivity. *Tospovirus* is the only genus in the virus family Bunyaviridae that infects the plants and is transmitted specifically by insects in the order Bunyavirales [1]. TSWV is transmitted in a persistent and propagative manner through the small insects *Frankliniella occidentalis,* commonly known as western flower thrips [2]. TSWV replicates in the midgut and salivary glands of the thrips without showing any significant pathogenicity to the former. TSWV contains three single-stranded RNA molecules as genetic material, named L (large), M (medium) and S (small) of which L is minus sense whereas M and S are ambisense [1]. TSWV is one of the deadliest *Tospoviruses* with a host range of more than 1000 plant species including tomato, tobacco and groundnut among others.

Tomato fruits have been considered as the principal component of the human diet and the primary source of a plethora of vitamins and minerals. Because of the huge demand and consumption of tomato fruits around the globe, it has been ranked as the ninth most cultivated agricultural plant with an annual production of 159 million tons per year [3] and the annual loss of tomato because of the TSWV is huge. Some of the common symptoms of the TSWV include necrotic leaves and ring spots and black streaks on the stems, however, specific symptoms vary from host to host. Particularly on tomato, mottling of immature fruits, light green rings and unique orange and red discoloration patterns have been reported on the mature fruits, thereby reducing their economic importance and further consumption. Therefore, it is extremely important to control this deadly virus, however, the major bottleneck is lack of the information underlying the TSWV-thrips and TSWV-tomato interaction [1].

Efforts have been made to identify the TSWV responsive proteins in thrips both at transcriptome [4,5,6] and proteome levels [7,8]. However, little effort has been given to understanding the molecular mechanism of TSWV-induced responses in tomato [1]. To the best of our knowledge, there is no report on the proteome analysis of tomato plants in response to TSWV infection which is required to understand the TSWV–tomato interaction in greater detail. Here, we are reporting a proteomic analysis of the tomato leaves in response to TSWV inoculation. Altogether, we are reporting 1022 TSWV modulated proteins (292 from resistance and 730 from susceptible cultivar) which can be used as potential candidates for the breeding programs aiming to develop the TSWV-resistant tomato plants.

## 2. Results

### 2.1. TSWV Infection and Samples Validation

**Figure 1:**
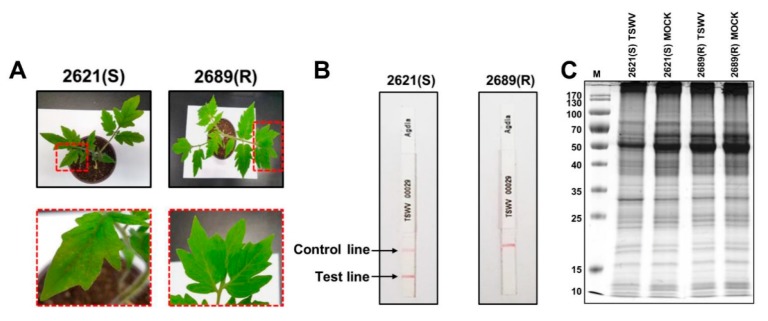
Validation of the samples. (**A**) Leaf morphology of susceptible (S) and resistant (R) cultivars of tomato after tomato spotted wilt virus (TSWV) inoculation. (**B**) ImmunoStrips showing successful infection of TSWV in the case of the susceptible cultivar. (**C**) SDS-PAGE of the isolated proteins from mock and TSWV infected leaves of tomato of susceptible (S) and resistant (R) cultivars.

Being one of the deadliest viruses, TSWV is a major threat to tomato productivity. Yet, the molecular pathways and signaling components affected by the TSWV infection in tomato leaves are still elusive, especially at the protein level. Here, we used a tandem mass tags (TMT)-based quantitative proteomic analysis to elucidate the TSWV induced signaling mechanism in tomato leaves. Tomato cultivars, namely 2689 and 2621, were selected and used respectively as TSWV resistant and susceptible cultivars. Susceptible cultivars showed clear signs of TSWV infection on the leaves after 3 days of TSWV inoculation while no such symptoms were observed in the case of the resistant cultivar (Figure 1A). Moreover, TSWV infection on the tomato leaves was further confirmed by the ImmunoStrips containing monoclonal antibodies against a TSWV-specific protein. Similar to the results observed morphologically, ImmunoStrips results showed successful TSWV infection in susceptible cultivar while no infection was observed in the case of the resistant cultivar (Figure 1B). The SDS-PAGE analysis of the isolated proteins from mock and TSWV-inoculated samples showed clear separation of proteins and some variations in the gel profiles of mock and TSWV-inoculated samples, including degradation of RuBisCO large subunit (Figure 1C).

### 2.2. High-Throughput Quantitative Proteome Analysis

**Figure 2:**
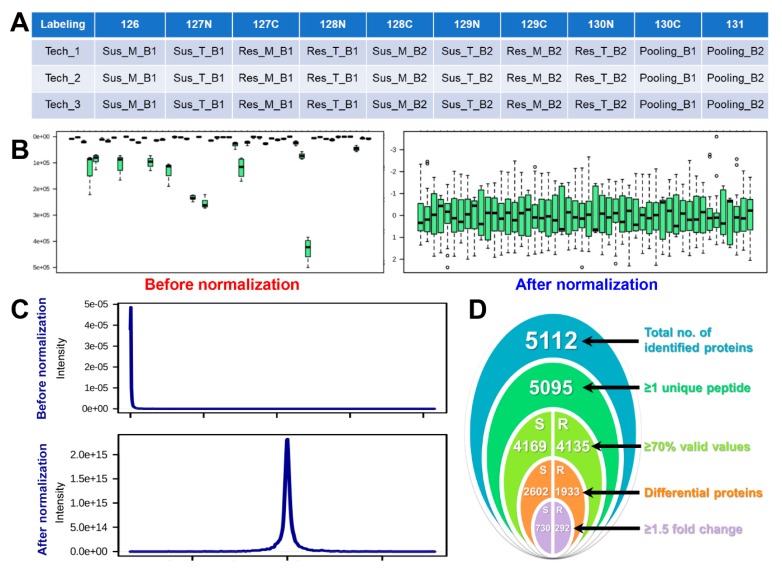
Data normalization and protein identification. (**A**) Table showing the labeling of different biological and technical replicates of the samples with 10-plex tandem mass tags (TMT)-kit. (**B**) Box plots and (**C**) showing protein intensity before and after normalization of proteins by the internal reference scaling (IRS) method. (**D**) Venn diagram showing the number of identified and differential proteins in susceptible and resistant cultivars of tomato after TSWV infection.

**Figure 3:**
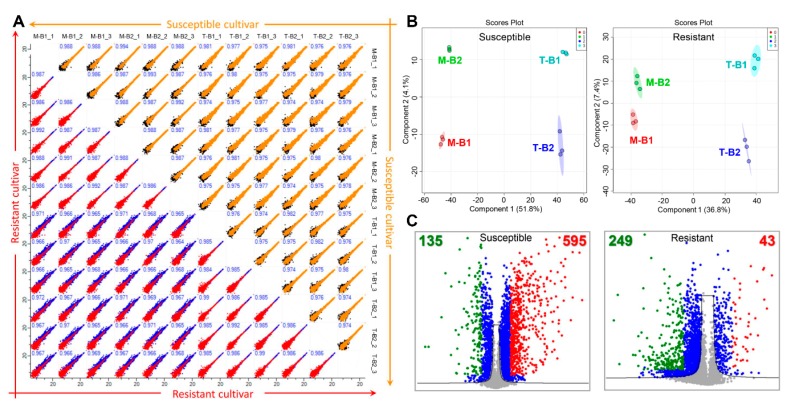
Statistical analysis of the identified proteins using Perseus and MetaboAnalyst software. (**A**) Multi-scatter plots of the identified proteins from susceptible and resistant cultivars identified proteins with the Pearson Correlation coefficient values. (**B**) Partial least squares-discriminant analysis (PLS-DA) plots showing a clear separation of mock and TSWV treated samples in susceptible and resistant cultivars. (**C**) Volcano plots showing significant proteins (marked by blue color) and 1.5-fold increased and decreased proteins in response to TSWV infection, marked in red and green colors respectively.

A TMT-based labeling approach was used for the proteome analysis where ten isobaric tags were labeled with different samples and replicates as highlighted in Figure 2A. Isolated proteins from two biological and three technical replicates were labeled with TMT-10 plex kit and pooled peptides were fractionated into 12 fractions by an in-house developed basic pH reverse phase chromatography [9]. For the normalization of TMT data, an internal reference scaling (IRS) method was adopted as described previously (Figure 2B) [10]. As per the IRS method, data normalization was carried out at two levels; first among the biological replicates of the same sample within a 10-plex TMT kit and second among the three technical replicates represented by separate MS runs or among different 10-plex TMT kit [11] (Figure 2C). This TMT-based approach led to the identification of a total of 5512 protein groups of which 17 showed less than one unique peptide (Figure 2D and Supplementary Table S1). These 17 protein groups were considered low-class identifications and were not used for the downstream analysis. Out of the remaining 5095 proteins, susceptible and resistant cultivars showed 4169 and 4135 protein groups with 70% valid values and were used for the statistical analysis (Figure 2D). In order to examine the reproducibility of the obtained TMT-data among different biological and technical replicates of the same sample, multi-scatter plots were generated using Perseus software. Scatterplots of the same samples showed a typical non-uniform spread, suggesting low accuracy and reproducibility for the peptides which are closer to the background level [12]. Pearson correlation coefficients of different replicates of the same samples were more than 0.980, indicating a high degree of correlation among different replicates of the same samples (Figure 3A).

### 2.3. Multivariate Analysis and Functional Annotation of the TSWV Responsive Tomato Proteins

**Figure 4:**
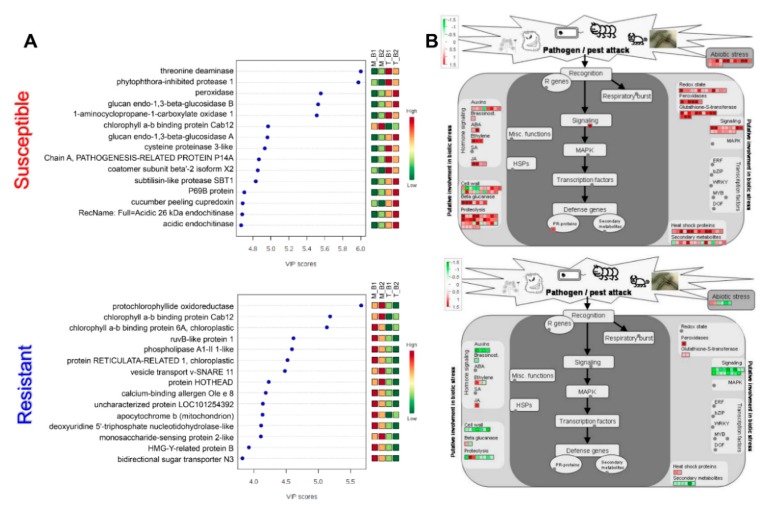
(**A**) Variable importance in the projection (VIP) scores plots showing the top 15 proteins with their VIP scores with the highest changes in abundances in response to TSWV inoculation. (**B**) MapMan biotic stress overview category showing proteins involved in the defense mechanism marked by the red and green color scheme. The upper panel shows results from susceptible cultivar while the lower panel shows results from resistant cultivar. Abbreviations: M_B1: Mock biological replicate 1; M_B2: Mock biological replicate 2; T_B1: TSWV treated biological replicate 1; T_B2: TSWV-treated biological replicate 2.

Partial least squares-discriminant analysis (PLS-DA) of control and virus treated samples of both susceptible and resistant cultivars were separated at component 1 accounting for 51.8% and 36.8% variations, respectively (Figure 3B). In addition, different biological replicates of the same sample were separated in component 2 which accounts for 4.1% and 7.4% of total variations in case of susceptible and resistant cultivars respectively (Figure 3B). Student’s *t*-test controlled by Benjamini Hochberg FDR < 0.05 was applied for the identification of significantly modulated proteins, resulting in the identification of 730 and 292 proteins showing more than 1.5-fold change in abundance in virus-infected samples as compared to control in susceptible and resistant cultivars respectively (Figure 2D and Supplementary Table S1). Of 730 differential proteins in susceptible cultivar, 595 and 135 showed increased and decreased abundances respectively while in case of resistant cultivar, 43 increased and 249 decreased proteins were observed (Figure 3C and Supplementary Figure S1). The variable importance in the projection (VIP) plots generated to identify the top 15 differential proteins in TSWV mediated stress response in tomato (Figure 4A).

### 2.4. Functional Annotation of the Identified Proteins

MapMan analysis of the differential proteins showed a clear up-regulation of the biotic-stress responsive proteins in susceptible cultivar while resistant cultivar showed fewer changes in response to TSWV inoculation (Figure 4B). In the case of primary metabolism overview, the majority of the proteins in susceptible cultivars were upregulated while that of resistant cultivar were downregulated upon TSWV inoculation (Supplementary Figure S2). In the cellular response overview category, only heat stress-responsive proteins were found to be upregulated in the resistant cultivar while susceptible cultivar showed increased abundance of proteins majorly associated with all the categories including abiotic stress, redox regulation, cell division, cell cycle and development (Supplementary Figure S2). In the case of enzyme families, resistant cultivar showed increased abundance of only Glutathione-S-transferases, peroxidases and β-1,3-glucan hydrolases while susceptible cultivar showed increased abundance of almost all the enzyme families mapped. In addition to MapMan, KEGG analysis was carried out and abundance patterns of the identified proteins were mapped on KEGG pathways using PathView, an R-based program [13]. KEGG pathway analysis showed that proteins related to the galactose metabolism, pyruvate metabolism, pentose phosphate pathway, TCA cycle, C3 cycle and amino acid metabolism were majorly increased in the case of susceptible cultivar while these remain majorly unaffected in case of the resistant cultivar. In the case of the MAP kinase signaling pathway, the majority of the MAP kinases either remained unaffected by TSWV infection or showed decreased abundance in case of resistant cultivar except for MPK1/4 which showed increased abundance. In the case of susceptible cultivar, the majority of these MAP kinases showed increased abundance in response to TSWV infection (Supplementary Figure S3). In the case of plant–pathogen interaction, proteins related to the PAMP-triggered immunity (PTI) responses including calcium-dependent protein kinase (CDPK), calmodulin (CaM) and respiratory burst oxidase homolog (RBOH) showed increased abundance in susceptible cultivar whereas a decreased abundance of these proteins was observed in resistant cultivar. In addition to these, mitogen-activated protein kinase kinase kinase 1 (MEKK1), mitogen-activated protein kinase kinase 1 (MKK1/2), glycerol kinase (NHO1), and heat shock protein 90 (HSP90) showed increased abundance in susceptible cultivar while these were majorly remained either unaffected or showed decreased abundance in the resistant cultivar (Supplementary Figure S4). Moreover, pathogenesis-related protein 1 (PR1) and enhanced disease susceptibility 1 protein (EDS1) showed increased abundances in both the cultivars while somatic embryogenesis receptor-like kinase 4 (SERK4), also referred as brassinosteroid insensitive 1-associated receptor kinase 1 (BAK1) showed no change in susceptible cultivar and was downregulated in the resistant cultivar following TSWV inoculation.

## 3. Discussion

Tomato is one of the most economically important plants and its productivity is constrained by several factors including abiotic and biotic stresses. Among the biotic stresses, TSWV is one of the major factors limiting tomato productivity around the globe. However, the molecular mechanism underlying the TSWV infection in tomato is currently elusive. Here, we used a TMT-based quantitative proteomics approach to identify the TSWV responsive proteins in susceptible and resistant cultivars of tomato. A contrasting difference in TSWV responses in the susceptible and resistant cultivars of tomato was observed. While 17.5% proteome of susceptible cultivar changed in response to TSWV infection, only ~7% proteome of the resistant cultivar was found to be TSWV responsive.

### 3.1. TSWV Impaired light Reactions of Photosynthesis

TSWV inoculation resulted in a decreased abundance of proteins involved in the tetrapyrrole synthesis and light reactions. Decreased abundances of two isoforms of magnesium-chelatase and three isoforms of protochlorophyllide reductase were observed in the susceptible cultivar upon TSWV infection. Magnesium-chelatase catalyzes the first and the rate-limiting step of chlorophyll biosynthesis by inserting Mg^2+^ into protoporphyrin IX [14] while protochlorophyllide reductases are involved in the conversion of protochlorophyllide to chlorophyllide, immediate precursors of chlorophyll [15]. As chlorophyll is the primary pigment involved in harvesting the sunlight, decreased abundances of these enzymes suggest overall downregulation of light reactions. Additionally, decreased abundances of different subunits and isoforms of light-harvesting complex, phosphoglycerate kinase, glyceraldehyde 3-phosphate dehydrogenase a subunit 2 and chlorophyll-a,b-binding proteins were also observed, further indicating downregulation of light reactions of photosynthesis. Inhibition of photosynthesis, mainly light reactions, has previously been shown in response to viral infections. A downregulation of photosynthetic electron transport in photosystem II (PSII) was reported in *Nicotiana benthamiana* infected with different strains of pepper and paprika mild mottle viruses [16]. Moreover, it was observed that coat proteins of some of the viruses such as tobacco mosaic virus, directly interact with PSII proteins and inhibit its functions [17]. Conversely, it has also been suggested that plants turn off the photosynthesis to invest more on respiration and defense-related processes [18].

### 3.2. Upregulation of Sugar and Amino acid Metabolism Pathways by TSWV

Apart from the light reactions, other metabolic pathways including glycolysis, TCA cycle, electron transport chain and amino acid metabolism, among others, were found to be upregulated in response to TSWV infection. Fructose bisphosphate aldolase, one of the key enzymes of glycolysis, C3 cycle and gluconeogenesis was found to be increased in response to TSWV infection. The involvement of this particular enzyme has been well established in the case of abiotic stresses including heat [19], cold [20], drought [21], salt [22] and cadmium [23]. However, there is not much information available on the expression pattern of this enzyme in case of biotic stress. Although there are no reports on the modulation of this enzyme by the virus infection in particular, some reports highlighted its role in biotic stress tolerance against *Fusarium* [24]. In addition to this enzyme, different subunits of cytochromes were also found to be upregulated by TSWV inoculation. Functions of cytochromes in the biotic stress tolerance are well established. It has been shown that the suppression of a cytochrome P450 gene compromises the basal pathogen defense response of pepper plants [25]. In particular, an enhanced susceptibility of pepper plants to bacterial pathogens was observed upon suppression of the cytochrome P450 gene. It was suggested that cytochrome P450 may mediate the plant defense responses through salicylic acid and ABA signaling pathways [25]. Yet another similar report suggested that the cytochrome P450 CYP82D regulates systemic cell death in cotton by modulating the jasmonate biosynthesis pathway [26]. Interestingly, here we also observed an upregulation of the enzymes involved in jasmonate biosynthesis, indicating the functioning of a similar mechanism of regulation of systemic cell death in tomato leaves in response to TSWV. Apart from these specific roles of the above enzymes in plant defense, it has been proposed that the overall upregulation of primary metabolism modulates signal transduction cascades culminating into plant defense responses [27].

### 3.3. Secondary Metabolism was Activated in Response to TSWV Infection

In the case of secondary metabolism, increased abundances of some of the key enzymes including phenylalanine ammonia-lyase, 4-coumaroyl-CoA synthase, naringenin-chalcone synthase, and dihydroflavonol-4-reductase were observed, indicating TSWV induced accumulation of secondary metabolites which may play defensive roles. In addition to these key enzymes, an increased abundance of two isoforms of anthranilate N-hydroxycinnamoyl/benzoyltransferase 3 was also observed. This enzyme has been reported to catalyze the first step of phytoalexin biosynthesis incarnation and was only induced in response to fungal elicitors [28]. Phytoalexins are a group of antimicrobial compounds including terpenoids, glycosteroids and alkaloids that are synthesized only in response to pathogen attack [29]. These phytoalexins play crucial roles in plant defense by inhibiting the growth and reproduction of invading pathogens. Although the functions of phytoalexins in viral defense are still elusive, few reports have highlighted their roles in viral infections. It was reported that a terpenoid phytoalexin plays a critical role in basal defense against *Potato virus X* in *N. benthamiana* as silencing of the phytoalexin synthesis genes attenuated the plant’s resistance to invading virus [30]. Furthermore, two isoforms of strictosidine synthase-like proteins were also found to be upregulated in response to TSWV infection. Strictosidine synthase is involved in the production of strictosidine which is a precursor for monoterpene indole alkaloids including quinine, camptothecin, ajmalicine, serpentine, vinflunine, vinblastine and vincristine [31]. Although pathways for strictosidine biosynthesis are not present in tomato and other members of the Solanaceae family, strictosidine synthase-like proteins have been identified in almost all the multicellular organisms including animals. In Arabidopsis, upregulation of strictosidine synthase-like genes was observed in response to exogenous treatment of salicylic acid, methyl jasmonate and ethylene [32]. In addition, wounding stress and inoculation of *Alternaria brassicicola* and cucumber mosaic virus also resulted in the upregulation of the strictosidine synthase-like genes [32], indicating their critical functions in plant defense signaling.

### 3.4. Fine-Tuning of the Redox Status of the Cells

Reactive oxygen species (ROS) burst with a transient increase in the apoplastic ROS concentrations is one of the earliest events in response to an invading pathogen and ROS so produced, especially H_2_O_2_, participates in several cellular events including cell signaling regulation and programmed cell death (PCD) [33]. ROS burst is mediated by the activity of a plasma membrane-localized NADPH oxidase (respiratory burst oxidase homolog; RBOH), amine oxidases and peroxidases [34]. Interestingly, in this study, the upregulation of RBOH and six isoforms of peroxidases was observed here in response to TSWV infection. However, as ROS in higher concentrations for longer durations are toxic to the cells, these must be quickly detoxified which is carried out by the activity of a series of enzymes including ascorbate peroxidase (APx), superoxide dismutase (SOD), monodehydroascorbate reductase (MDHAR), dehydroascorbate reductase (DHAR) and catalase (CAT). Here, we observed an increased abundance of 13 proteins involved in ROS scavenging and includes an APx, two isoforms of Fe-SOD, Mn-SOD, MDHAR, DHAR and CAT, suggesting active detoxification of ROS after the ROS burst. It is well known that a delicate balance is required between the ROS production and ROS scavenging during pathogen attack and maintenance of this balance is one of the determining factors of the plant’s performance under stress conditions [34,35].

A total of 13 isoforms of Glutathione-S-transferases (GST) were also identified here and interestingly, all of these isoforms showed an increased abundance in response to TSWV infection in the susceptible cultivar. With the help of nucleophilic glutathione, GST proteins are involved in the vacuolar sequestration of electrophilic xenobiotics that are formed during pathogen attack. Moreover, some of the recent reports have highlighted the functions of GSTs in the regulation of hypersensitive responses (HR) during plant-virus interaction [36]. It was shown that the activity of antioxidant enzymes, including GST, decreases after the tobacco mosaic virus, however, the activities were markedly increased following HR development [37]. Furthermore, some of the GSTs function as glutathione peroxidase and thus detoxify ROS and toxic lipid hydroperoxides that are produced during pathogen invasion [36], suggesting that GSTs play a pivotal role in plant defense. Likewise, the upregulation of all the identified GSTs here suggests their key roles in the plant’s defense against TSWV infection.

Protein disulfide isomerase (PDI) proteins are localized in the endoplasmic reticulum in eukaryotes and are involved in the rearranging of the disulfide bonds for proper folding of the misfolded proteins during normal growth and stress conditions. Here, we observed an increased abundance of five isoforms of PDI in response to TSWV infection. Rapid induction of the PDI genes has previously been observed during biotic stress in wheat in response to a hemibiotrophic fungal pathogen *Mycosphaerella graminicola*, indicating their involvement in plant defense [38]. In addition to their routine functions as molecular chaperons, PDI proteins have also been shown to regulate the PCD in plants [39]. PCD is the self-destruction of the infected cells and is one of the ways to check the transfer and multiplication of the invading pathogens [40]. It was shown that one PDI5 in Arabidopsis is involved in the regulation of PCD by physically interacting with the cysteine proteases [41].

### 3.5. Activation of the Stress Response and Phytohormones Signaling

A transient increase in the calcium ion (Ca^2+^) concentrations and activation of MAP kinase signaling together with ROS burst are some of the earliest events during pathogen infection. Here, we observed an increased abundance of calcium-dependent protein kinase (CDPK) and calmodulin (CaM) after TSWV infection in susceptible cultivar while these were majorly downregulated in the resistant cultivar. Both CDPK and CaM are considered as Ca^2+^ sensors and their functions in the plant immune signaling, especially during PAMP triggered immunity is well established [42]. It has been shown that overexpression of CaM-4/-5 in soybean resulted in enhanced tolerance against a wide spectrum of pathogens including viruses [43]. Moreover, the direct involvement of CaM proteins in the suppression of virus-induced gene silencing has also been reported in tobacco [44,45]. In addition, a tobacco CaM, termed as rgs-CaM (regulator of gene silencing), has been shown to play a role in systemic acquired resistance against cucumber mosaic virus [46]. It was hypothesized that rgs-CaM acts as a receptor for both viral RNA silencing suppressors and Ca^2+^ influx and activates the salicylic acid signaling pathways following virus infection [42,46].

CDPKs also function upstream of RBOH and CDPK mediated phosphorylation of RBOH is crucial for ROS burst [47]. Interestingly, an increased abundance of both of these proteins was observed upon TSWV infection, suggesting a pivotal role of these proteins and ROS burst in the activation of plant defense signaling against TSWV. In addition, increased abundances of several MAP kinases were observed in the susceptible cultivar following TSWV infection while these remain majorly unaltered in the resistant cultivar, indicating that increased abundances of these proteins were specific to TSWV infection. In particular, an increased abundance of MEKK1, MKK1/2 and MPK3/6 suggesting activation of late plant defense response, cell death and H_2_O_2_ production, and production of pathogenesis-related (PR) proteins including basic endochitinase-B and PR1 [48]. Moreover, MAP kinases also participate in the ABA signaling and it has been shown that MPK1/2 functions downstream of SNF1-related protein kinase-2 (SnRK2), a member of plant-specific serine/threonine kinases [49]. SnRK2 positively modulates the ABA-signaling by phosphorylating MEKK17/18 and an increased abundance of SnRK2 was observed in response to TSWV infection, indicating activation of ABA-signaling. In addition to ABA, proteins related to ethylene, salicylic acid and jasmonate signaling were also found to be increased. In the case of jasmonate, we observed an increased abundance of enzymes involved in jasmonate biosynthesis. A total of four enzymes including lipoxygenase 1, allen oxide cyclase 3, 12-oxophytodienoate reductase-2 (OPR2) and OPR3 showed increased abundance, indicating TSWV infection induces jasmonate biosynthesis in tomato. It has been shown that a balance of jasmonate and salicylic acid is a determining factor for resistance to tobacco mosaic virus in tobacco [50]. It was shown that the silencing of either jasmonate or salicylic acid biosynthesis or signaling components results in enhanced susceptibility of tobacco against the tobacco mosaic virus [51]. Moreover, an accumulation of jasmonate at the early stages and salicylic acid at the later stages of virus infection were observed in tobacco [51], further confirming that the delicate balance of these two phytohormones is a determining factor of a plant’s resistance against an invading virus.

## 4. Materials and Methods

### 4.1. Plant Growth and TSWV Treatment

One week after germination, the tomato seedlings were transplanted into a 32-plug tray (6 cm in diameter by 6.5 cm in height) and maintained in a growth room at 24 ± 1 °C. A 16-h photoperiod was applied with a photon flux of about 600 µmoL m^−2^ s^−1^ PAR above the canopy. Air relative humidity was maintained at 70%. At the two-true-leaf stage, cotyledons were inoculated with TSWV diluted in PBS buffer by application onto upper leaves abraded with 400-mesh carborundum (Sigma, MO, USA), followed by gentle rubbing to spread the inoculum. Mock inoculations received the same treatment, but PBS buffer was used instead of virus inoculum. For proteome profiling, unwounded systemic leaves of the same developmental stage from six plants were harvested at 14 days after treatment. TSWV ImmunoStrip tests (Agdia, IN, USA) were performed to test the infection of the virus into tomato plants according to the manufacturer’s protocol before leave harvest. Briefly, 0.15 g tissue was placed in a sample extraction bag containing Sample Extraction Buffer, and the tissue was macerated with homogenizer into the emulsion in the buffer. The ImmunoStrips were inserted into the solution and the infected leaf samples were flash-frozen in liquid nitrogen and stored at −80 °C until protein isolation.

### 4.2. Protein Extraction, TMT Labeling and Q-Exactive MS Analysis

Protein extraction, trypsin digestion and TMT labeling were carried out as described previously [10]. In brief, proteins from the control and TSWV infected leaves of susceptible and resistant cultivars were extracted using Tris-Mg/NP-40 buffer (0.5 M Tris-HCl (pH 8.3), 2% *v*/*v* NP-40, 20 mM MgCl_2_) followed by TCA/Acetone precipitation of the proteins [12]. Trypsin digestion by the FASP method [52], TMT-labeling [9] and MS analysis [53,54] were carried out as described previously. The obtained proteomics data have been deposited to the ProteomeXchange Consortium via the PRIDE [55] partner repository with the dataset identifier PXD017250.

### 4.3. LC-MS/MS Data Analysis

The acquired MS data were analyzed with MaxQuant (ver. 1.5.3.30) [56]. MS/MS spectra were searched with the integrated Andromeda search engine against the tomato protein database (45,232 entries) downloaded from NCBI and 248 common contaminant proteins. Trypsin specificity was required and a maximum of two missed cleavages was allowed [57]. Carbamidomethylation of cysteine residues was set as fixed modification while TMT-labeled N-term, oxidation of methionine and protein N-terminal acetylation were set as variable modifications. A minimum peptide length of six amino acids was specified and “match between runs” (MBR) was enabled with a matching time window of 0.7 min. The allowed mass deviation was 4.5 ppm for peptides and 20 ppm for fragments [10]. Peptide-spectrum-matches and proteins were retained if they were below a false discovery rate of 1%. Statistical analyses, hierarchical clustering analysis (HCL) and principal component analysis (PCA) were carried out using Perseus software (ver. 1.5.8.5) [58]. Hits were only retained if they were quantified in at least 70% of the total replicates. Missing values imputation of protein intensities were performed from a normal distribution (width: 0.3, downshift: 1.8). Student’s *t*-test controlled by Benjamin Hochberg FDR < 0.05 was applied to identify the significant differences (≥1.5-fold change) in the protein abundance [59].

### 4.4. Bioinformatics Analysis

The MapMan program, version 3.6.0 RC1, was used for pathway analysis [60]. Proteins fold change values were transformed into Log_2_ fold change, and their means were calculated. These non-redundant proteins or genes were classified into MapMan BINs and their annotated functions were visualized using the MapMan program. Partial Least Squares Discriminant Analysis (PLS-DA) was performed using MetaboAnalyst [61]. KEGG pathway mapping was carried out using the PathView program [13].

## 5. Conclusions

**Figure 5:**
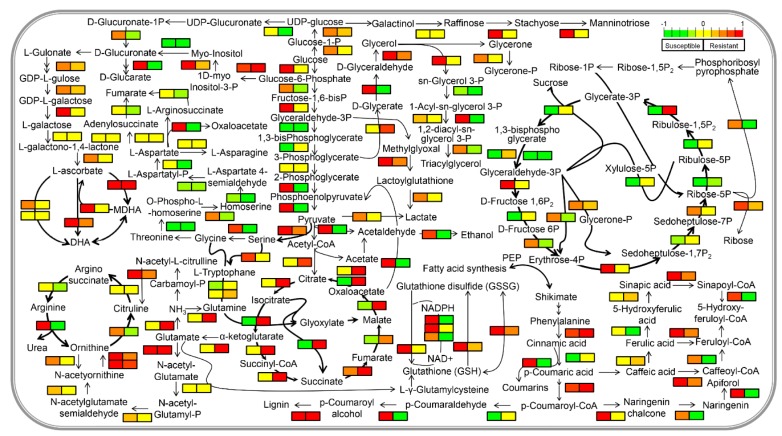
Mapping of identified proteins in respective metabolic pathways to visualize the overall changes in the metabolism in response to TSWV infection in resistant and susceptible cultivars of tomato. The abundance pattern of the proteins is shown by the green–yellow–red color scheme. The first panel shows the results from susceptible cultivar while the right panel shows the results from the resistant cultivar.

Deciphering the molecular mechanism of TSWV induced signaling in tomato is crucial in order to control this deadly virus. The work reported here is a first approach to identify the protein components induced by TSWV in tomato that resulted in the identification of 1022 proteins showing more than 1.5-fold change in response to TSWV infection. A global picture of the changes observed (Figure 5) showed that TSWV infection results in an overall downregulation of tetrapyrrole synthesis and light reactions of photosynthesis while the upregulation of primary and secondary metabolism was observed. Calcium signaling seems to play a crucial role in plant defense against TSWV as several components of calcium signaling pathways including CDPK and CaM were found to be upregulated following TSWV infection. Similarly, several proteins related to the jasmonate biosynthesis were also found to be upregulated, suggesting jasmonate production following TSWV infection which could play a critical role in the tomato-TSWV interaction. Furthermore, several other proteins including MAP kinases, GSTs and peroxidases, among others, highlighting their key functions in plant defense against TSWV. Future efforts could be on the validation of these identified proteins using a combination of biochemical and molecular biology approach to establish the roles of these proteins in TSWV-tomato interaction.

## Supplementary Materials

The following are available online at https://www.mdpi.com/2223-7747/9/3/290/s1, Figure S1: Hierarchical clustering analysis showing differential proteins marked by the red and green color scheme representing up- and down-regulated proteins respectively. The first panel shows the results from TSWV infected to treatment in the susceptible cultivar while the right panel shows the results from the TSWV inoculated in the resistant cultivar; Figure S2: MapMan metabolism overview category showing proteins involved in the defense mechanism marked by the red and green color scheme. The first panel shows the results from susceptible cultivar while the right panel shows the results from the resistant cultivar; Figure S3: Functional annotation of the identified proteins in the MAP kinase signaling of the KEGG pathway. For showing the differential abundance of the identified and mapped proteins, intensity values of the identified proteins were mapped to respective pathways by PathView program. The expression pattern of the mapped proteins is marked by the Red and Green color scheme. The first panel shows the results from susceptible cultivar while the right panel shows the results from the resistant cultivar; Figure S4: Functional annotation of the identified proteins in the plant-pathogen interaction of the KEGG pathway. The expression pattern of the mapped proteins is marked by the Red and Green color scheme. The first panel shows the results from susceptible cultivar while the right panel shows the results from the resistant cultivar; Table S1: List of the identified and differentially modulated proteins from susceptible and resistant cultivars.
