# Supplementary material for: A TMT-Based Quantitative Proteome Analysis to Elucidate the TSWV Induced Signaling Cascade in Susceptible and Resistant Cultivars of Solanum lycopersicum
Source: Plants (Basel). 2020 Feb 26;9(3):290. doi: 10.3390/plants9030290 (PMC7154910; doi:10.3390/plants9030290)
Supplement: Supplementary file 1 [file plants-09-00290-s001.zip › Supplementary Figures.pptx]

## Slide 1
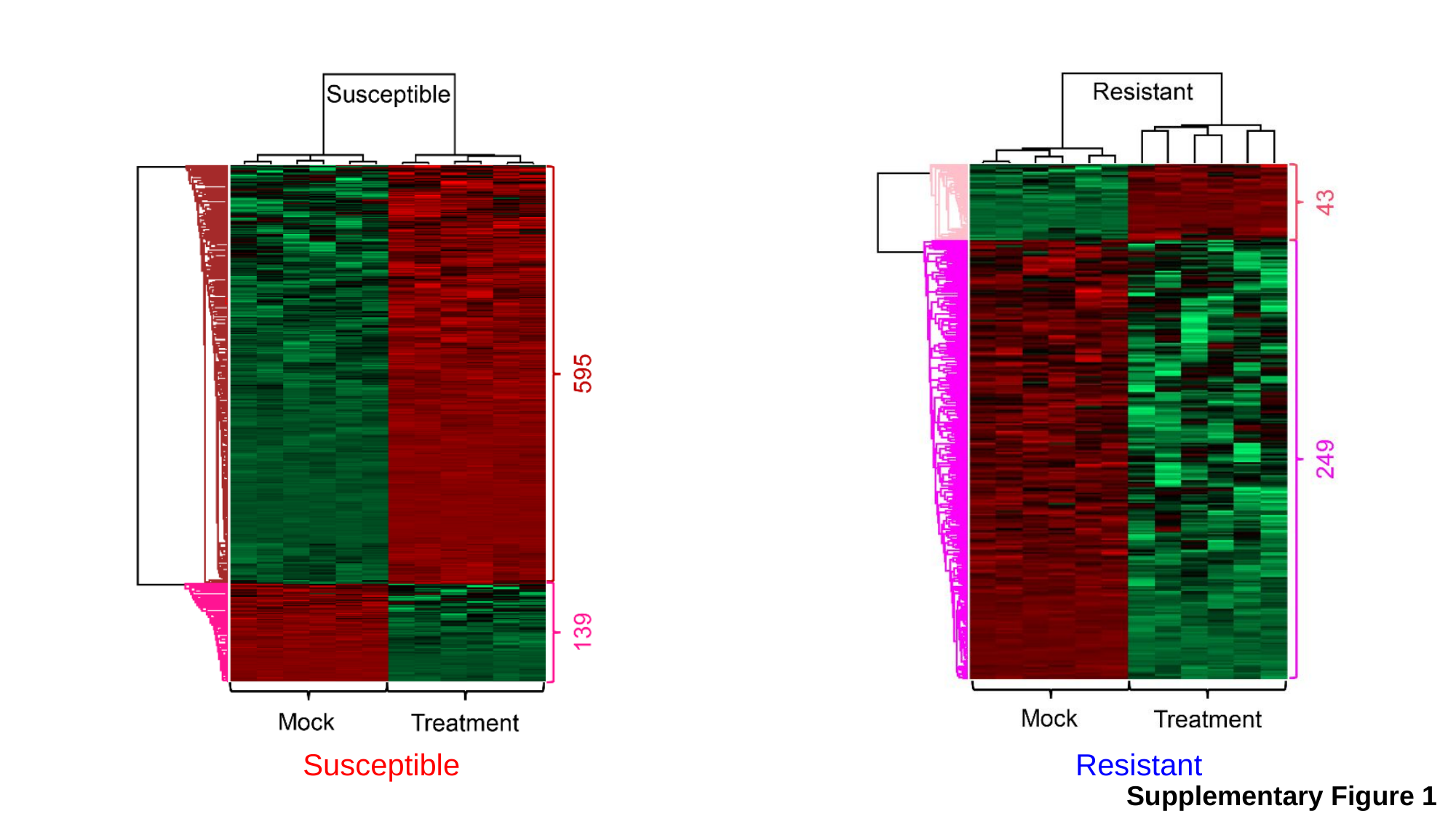

Susceptible
Resistant
Supplementary Figure 1

## Slide 2
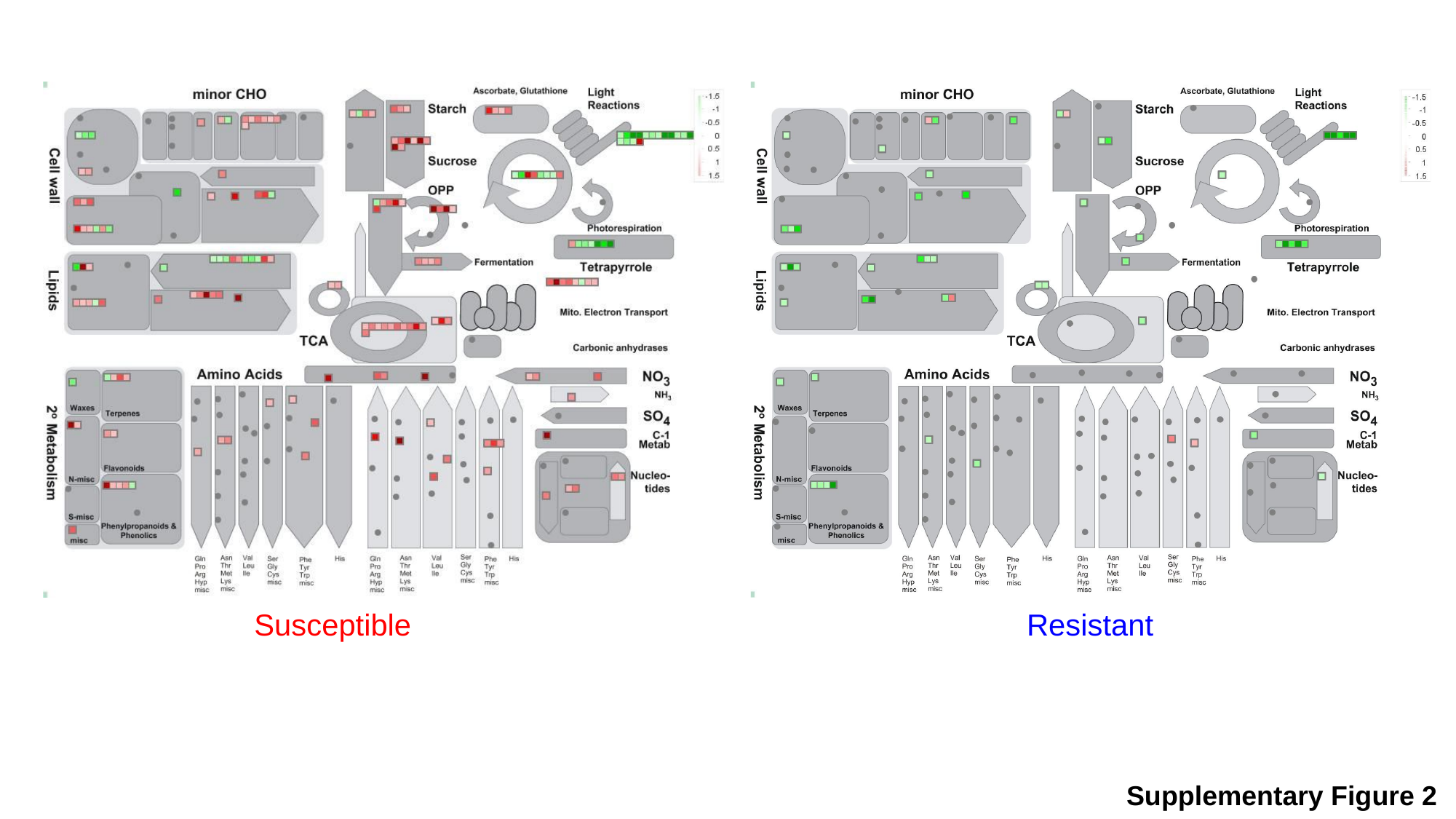

Susceptible
Resistant
Supplementary Figure 2

## Slide 3
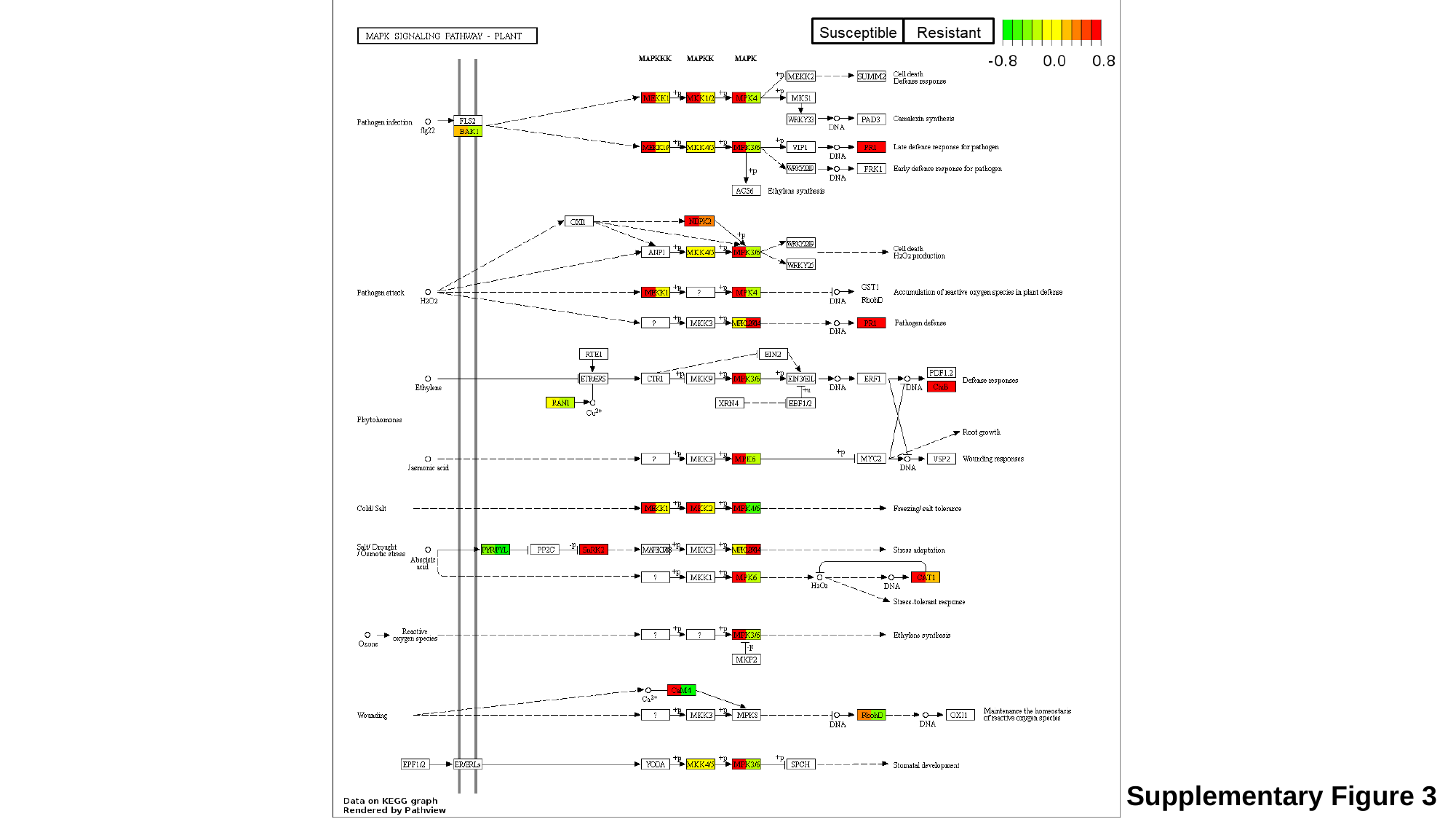

Supplementary Figure 3

## Slide 4
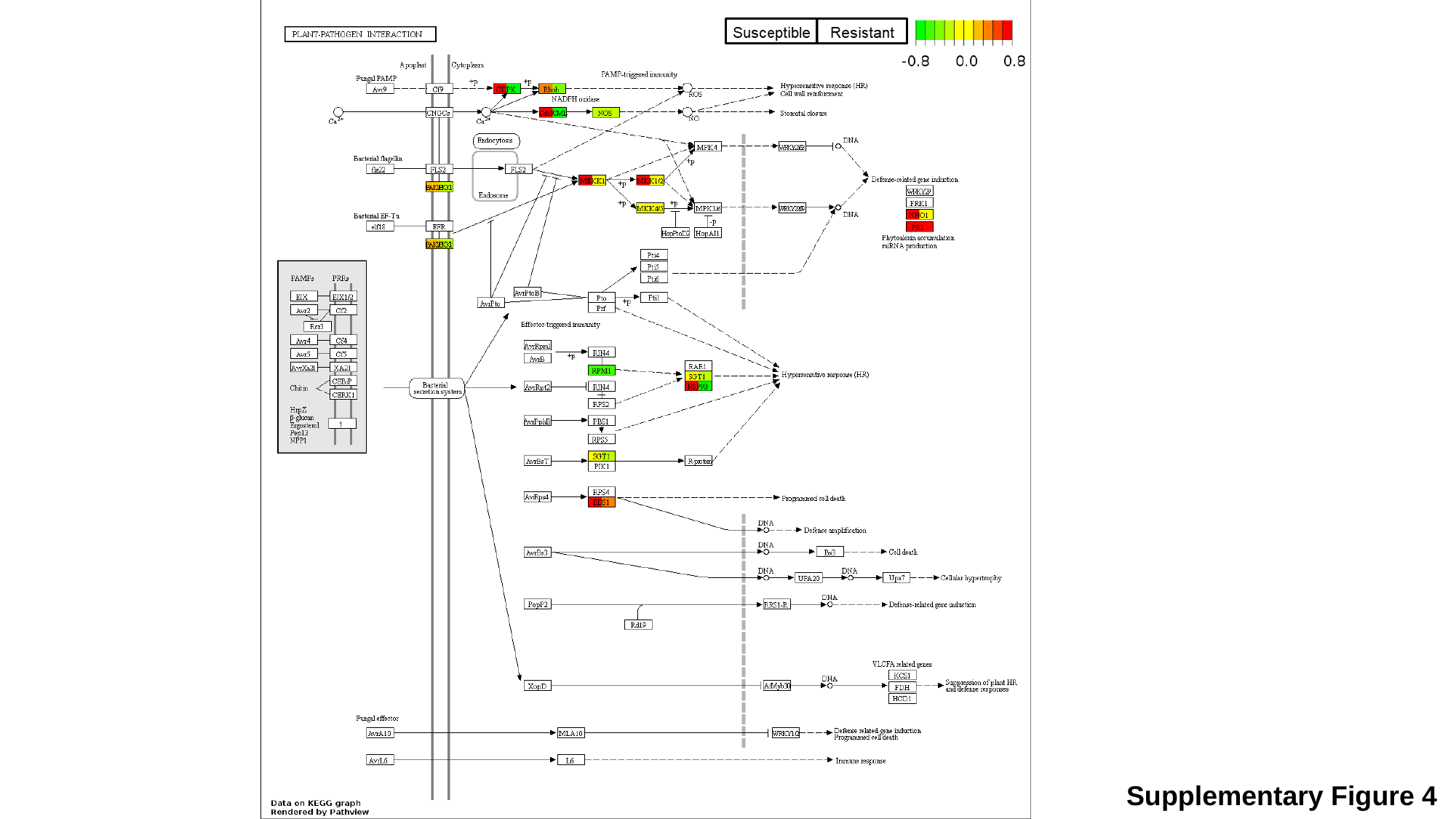

Supplementary Figure 4

## Slide 5
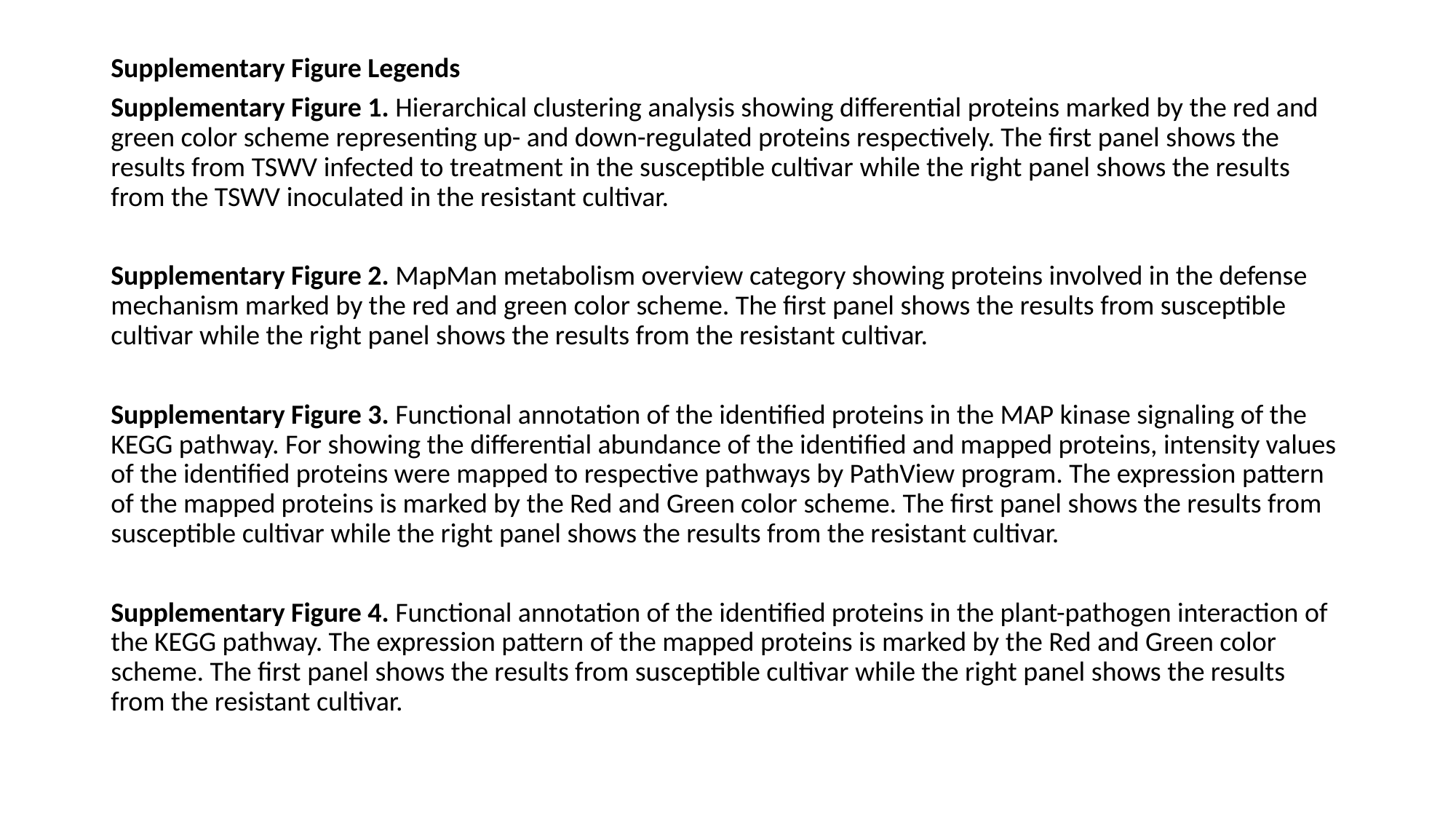

Supplementary Figure Legends
Supplementary Figure 1. Hierarchical clustering analysis showing differential proteins marked by the red and green color scheme representing up- and down-regulated proteins respectively. The first panel shows the results from TSWV infected to treatment in the susceptible cultivar while the right panel shows the results from the TSWV inoculated in the resistant cultivar.
Supplementary Figure 2. MapMan metabolism overview category showing proteins involved in the defense mechanism marked by the red and green color scheme. The first panel shows the results from susceptible cultivar while the right panel shows the results from the resistant cultivar.
Supplementary Figure 3. Functional annotation of the identified proteins in the MAP kinase signaling of the KEGG pathway. For showing the differential abundance of the identified and mapped proteins, intensity values of the identified proteins were mapped to respective pathways by PathView program. The expression pattern of the mapped proteins is marked by the Red and Green color scheme. The first panel shows the results from susceptible cultivar while the right panel shows the results from the resistant cultivar.
Supplementary Figure 4. Functional annotation of the identified proteins in the plant-pathogen interaction of the KEGG pathway. The expression pattern of the mapped proteins is marked by the Red and Green color scheme. The first panel shows the results from susceptible cultivar while the right panel shows the results from the resistant cultivar.
